# A Qualitative Exploration of the Experiences of Disclosing Non-Monogamy

**DOI:** 10.1007/s10508-025-03119-0

**Published:** 2025-03-24

**Authors:** Joel R. Anderson, Alena Bondarchuk-McLaughlin, Scarlet Rosa, Karen D. Goldschlager, D. X. Hinton Jordan

**Affiliations:** 1 Australian Research Centre in Sex, Health and Society, La Trobe University, NR6, Bundoora, Melbourne, VIC 3085 Australia; 2 School of Global, Urban and Social Studies, Royal Melbourne Institute of Technology, Melbourne, Australia; 3 School of Behavioural and Health Sciences, Australian Catholic University, Melbourne, Australia

**Keywords:** Non-monogamy, Consensual non-monogamy, Ethical non-monogamy, Disclosure, Polyamory

## Abstract

Non-monogamy refers to a set of relationship identities and structures that go against societal norms and expectations of “typical” relationships (i.e., monogamous heteronormative dyadic relationships). As such, non-monogamous individuals often conceal this identity and then need to engage in continuous decision-making processes regarding when and how to disclose, due in part to the structural barriers, stigma, and discrimination faced by this population. This study sought to explore the experiences of the disclosure of consensual (or ethical) non-monogamy and the factors impacting on the decision to disclose across a range of environments. Data on lived experiences were explored by conducting qualitative semi-structured interviews with 32 non-monogamous adults living in Australia. Based on grounded theory, four key themes were identified during the analysis of the data: (1) decisions around how and when to disclosure are complex, (2) responses to disclosure are typically negative, (3) structural barriers typically prevent disclosure, and (4) unless specifically trained, healthcare providers are typically uninformed about non-monogamy. These findings are discussed in relation to providing a base model of disclosure for future research to build upon and to enhance understanding of systemic and healthcare-related issues faced by this population. This research adds to the current understanding of factors impacting disclosure, with possible clinical implications and suggestions for future research discussed.

**Supplementary Information:**

The online version contains supplementary material available at 10.1007/s10508-025-03119-0.

## Introduction

Current research has estimated that approximately 5% of the population of the USA are engaged in relationships that are non-monogamous by mutual agreement (Scoats & Campbell, 2022). Non-monogamy is an umbrella term that is used to describe a range of relationship structures, practices, and identities that involve having (or being open to having) concurrent relationships with more than one other consenting adult, with the explicit awareness of all parties (Hamilton et al., 2021; Smith, 2017). Because of the awareness of all involved, this is often referred to as consensual or ethical non-monogamy (we note that the similarities and differences between these terms vary between individuals and across contexts). For those in non-monogamous relationships, the term can describe relationship structures or sexual practices within the relationship/s. For non-monogamous individuals, it can describe an identity, an orientation, or a preference to dating and sex. It is worth noting that an individual’s non-monogamous identity does not always align with their relationship configuration. For example, someone can identify as non-monogamous (or have preference for non-monogamy) and be in a relationship with one person or be single. Finally, non-monogamous relationship structures and practices are not fixed, and individual orientations toward relationships are fluid and often change across time (for discussions, see Cardoso et al., 2021; Gupta et al., 2024; Rubel & Burleigh, 2020).

Common non-monogamous relationship agreements include an open relationship (a relationship that does not limit its participants from engaging in romantic/sexual relationships with other people), *polyamory* (committed romantic relationships with multiple partners), *swinging* (consensual sex with other sexual partners in addition to a primary relationship), and several others (Balzarini & Muise, 2020; Hosking, 2013; Levine et al., 2018, see glossary of terms about non-monogamy in supplementary Table S1). While non-monogamous relationships are estimated to make up a small minority of relationship styles, the estimated occurrence rate is still considerable (and increasing, see Moors et al., 2017), yet vastly underrepresented in the literature on relationship styles.

Non-monogamous identities and relationship configurations have historically been widely misunderstood and stigmatized (Barker & Langdridge, 2010; Conley et al., 2013a, 2013b; Hutzler et al., 2016). For instance, Conley et al. () found that people idealized monogamy over non-monogamy and viewed monogamy through a morally superior lens. This is likely because alternative relationship styles may challenge the current heteronormative cultural ideas of family, marriage, and commitment. Despite evidence suggesting that non-monogamous and monogamous individuals experience equivalent average levels of relationship satisfaction (Conley & Piemonte, 2021; Flicker et al., 2021, see Anderson et al., 2025 for a meta-analysis), the evidence suggests that non-monogamous people experience stigma and structural barriers based on their relationship orientation, and that this prevents adequate healthcare and social protections, such as circumscribed insurance policies and legal protections which exclude non-monogamous relationships (Klesse, 2006; McCrosky, 2015). Adoption and fostering can also be difficult and sometimes even inaccessible to those who are non-monogamous. For example, in Australia (where the data presented in this paper were collected) The Adoption Act, 1984 (Vic) includes a relationship clause which specifies that the members of an adopting couple must not be in registered domestic relationships with any other person.

Stigma is known to contribute to both selective disclosure and complete non-disclosure for non-monogamous individuals (Thompson et al., 2020; Valadez et al., 2020). As a result, researchers have begun exploring the range of reasons that non-monogamy is not disclosed, particularly in healthcare settings. Manley et al. (2018) found that non-monogamous people chose not to disclose their relationship identities to others in their lives if they anticipated it might cause them subsequent harm (e.g., strain on their relationships, discrimination, or harassment). They also chose not to disclose to people who they felt may be incapable of absorbing or accepting the information, or when relationship information was deemed irrelevant or private (e.g., employment settings).

Concealment of non-monogamous relationships and a general lack of non-monogamy-related education may augment the failure of healthcare professionals to determine an accurate conceptualization of patients and result in the provision of inadequate care (Smith, 2017; Vaughan et al., 2019). Research conducted by Meyer (2003) provides evidence for the substantial and ongoing harm to mental health caused by the pervasive concealment of minority identities (i.e., minority stress theory; Meyer, 2003, see also Meyer & Frost, 2013; Nguyen et al., 2023). Although the aforementioned minority stress research was focused on sexual minority identities, this framework could be (and has been, see Moors et al., 2021a, 2021b; Witherspoon & Theodore, 2021) extended to include non-monogamous relationships. Additional in-depth research is required to gain a thorough understanding of the scope of stigmatization faced by this community and the barriers to disclosure of non-monogamy (Brewster et al., 2017).

The aim of this study was to examine the factors influencing disclosure decisions around non-monogamy. To test this aim, a series of qualitative interviews were conducted with non-monogamous individuals in order to build an evidence base around the experiences of selective disclosure and non-disclosure of non-monogamy, as well as other factors (such as stigma) that may contribute to the decision to disclose. During the interviews there was a focus on exploring experiences of disclosure across multiple settings, the affective responses of the disclose, and any consequences resulting from the disclosure.

## Method

### Participants

The sample was 32 self-identified non-monogamous Australian adults aged between 22 and 58 years (*M*_age_ = 34.42 years, SD = 9.19, see Table 1 for a detailed summary of the demographic characteristics of the sample). Participant eligibility criteria included self-identification as non-monogamous and being over 18 years old (relationship status was not a criteria). All participants lived in Australia and were tertiary educated, many at a post-graduate level (41%). Notably, all participants identified with a sexuality other than heterosexual, in keeping with previous research that found people with a minority sexuality identity to be more likely than heterosexual people to be non-monogamous (Scoats & Campbell, 2022; Sheff, 2019). Participants were recruited via targeted virtual advertising on social media with no offer of remuneration.

**Table 1:** Sample demographics for consensually non-monogamous people

Demographic category	Sub-categories	Percentage of sample %
Relationship identity	Non-monogamous	18.8
	Ethically non-monogamous	18.8
	Consensually non-monogamous	15.7
	Monogamish	9.4
	Relationship anarchist	9.4
	Polyamorous	9.4
	Committed to multiple intimate relationships	6.3
	Solo-polyamorous	3.2
	Polyfidelitous	3.1
	Polysexual	3.1
	Ambiamorous	3.1
Gender	Woman	37.5
	Man	25
	Gender non-binary	9.4
	Gender diverse	9.4
	Gender fluid	6.3
	Transfeminine	6.3
	Transmasculine	3.1
	Agender	3.1
Sexual orientation	Bisexual	40.6
	Pansexual	25.0
	Queer	18.8
	Mostly heterosexual	3.1
	Heteroflexible	3.1
Highest level of education	High school	12.5
	Bachelor course	31.3
	Postgraduate course	46.9
	Vocation	6.3
	PhD	3.1
Religious background	Spiritual	28.1
	Nil religion	28.1
	Atheist	18.8
	Buddhist	12.5
	Christian	6.3
	Islamic	3.1
	Wiccan	3.1
Cultural background	Anglo Australian/White	43.8
	European/White	25.0
	Multiracial	15.7
	Asian	9.4
	Middle Eastern	3.1
	Black	3.1
Living arrangement	With one partner	25.0
	Housemate/flatmate	21.9
	Split living between two partners/residences	21.9
	Solo	18.8
	With multiple partners in one residence	12.5
Children	No	81.3
	Yes (and live with)	12.5
	Yes (but do not live with)	6.3

Not all participants provided a response for all demographic categories

### Measures

This study used semi-structured interviews to explore what factors influenced decision making in the disclosure of non-monogamy. The data were collected via both face-to-face interviews and online interviews using the video conferencing platform Zoom. Participants were asked to provide demographic information including sex presumed at birth, gender identity, sexual identity, occupation, attained level of education, religious and cultural backgrounds, and residential arrangements.^1^

The interview schedule was comprised of a series of questions including: (1) How do you describe yourself with regard to your non-monogamous identity or relationship style? (2) When did it occur to you that you might be interested in non-monogamy and how did that emerge? (3) How openly do you disclose your non-monogamy? (4) Describe your experiences of disclosure of non-monogamy. Participants were asked follow-up questions where appropriate, including factors that contributed to the ease or difficulty of disclosure.

### Procedure

Prospective participants emailed the researcher and were sent a copy of the consent form, information sheet, and interview schedule in order to familiarize themselves with the purpose of the research before arranging an interview time. Interviews commenced with a reminder of confidentiality and participant rights, including the ability to withdraw from the study until de-identification had occurred. Following this, consent forms were signed and the interview proper commenced. Twelve interviews were completed face-to-face and 20 were conducted over Zoom™ (video conferencing software). All interviews were audio recorded with participant consent. After each interview, participants were given or emailed a debriefing form.

The recordings were reviewed by the research team and transcribed verbatim. Transcriptions were emailed to the participants to allow for revisions or additions and were then de-identified prior to analysis and the audio recordings deleted.

### Analysis Strategy

Data procurement occurred using an iterative process of collection, transcription, and analysis. Analyses were conducted using the qualitative analysis software NVivo 1.3 (Solutions, 2001). Data analyses were conducted using a two-step analytic process based on grounded theory prior to theme extraction and categorization. The first step was open coding, in which initial codes were assigned to data in small segments in order to generate a preliminary set of codes that serve as the foundation for deeper analysis. The second step was selective coding, in which the initial codes are refined and synthesized into coherent higher categories that represent core themes in the data. Grounded theory was chosen as a means of constructing an understanding of psycho-social experiences, noting that theoretical understandings were not driving the research question. The data were coded by two researchers—interviews from the first 2 participants were double coded to generate a code book, and then another 5 interviews were double coded to ensure a degree of inter-coder reliability, which together resulted in 21.9% of the data being coded (which is within the 10–25% of data units range as suggested by Campbell et al., 2013; O’Connor & Joffee, 2020). The remaining interviews were coded by the first and final authors (approximately half each). While the coding was being undertaken, the researchers made notes for points of ambiguity, uncertaintly, or confusion in the data, and these were discussed at team meetings. This allowed regular calibration of the coding process throughout.

The majority of the data presented in the analyses below are verbatim quotes from the data corps that represent the identified themes. At points, these quotes are supplemented with some quantitative comments about how frequently such quotes appeared in the data. These comments were included to indicate how prominent and common these experiences were for the participants.

Forrester and Sullivan (2018) acknowledged the importance of reflexive research due to the difficulty of truly objective qualitative research. To encourage reflexivity, memo writing was maintained throughout the analysis process. Through the process of self-reflection and discussion of themes among the research team, we believe subjectivity was reduced from within the analysis.

#### Positionality Statement

The research team have lived experience with being in and advocating for non-monogamous relationships. The members of the research team have experiences of being in relationships of different structures and configurations (including monogamous and a range of non-monogamous structures). We feel well-positioned to work on this project; however, we acknowledge that as White and cisgender researchers in relationships that our identities are very specific and often privileged, and that we cannot understand (and we do not represent) the full range of experiences of non-monogamy.

## Results

The completion time for the 32 interviews ranged from 26 to 67 min. Following the aforementioned strategy of data analysis, 154 codes were extracted from the data explaining participants’ experiences of disclosure. These codes were combined into clusters of similarity from which sub-themes and themes were identified.

Four key themes were extracted from the data: (1) Decisions around how and when to disclose are complex, (2) responses to disclosure are typically negative, (3) structural barriers typically prevent disclosure, and (4) unless specifically trained, healthcare providers are typically uninformed about non-monogamy. Each theme was explored and illustrated below using excerpts from the data (themes and sub-themes are presented in Table 2).

**Table 2:** Themes and associated sub-themes from data exploring decisions to disclose consensual non-monogamy

Themes	Associated sub-themes
Decisions around disclosure are complex	Privacy Energy conservation Risk mitigation Desire for genuineness and integrity Introduction of partners into life Enhance general representation of CNM Reduce assumptions of infidelity Anticipated stigma prevented disclosure
Responses to disclosure are typically negative	Observable discomfort No response after disclosure Misunderstanding nature of CNM Determining CNM is immoral Determining CNM is difficult or unpleasant Rejection of CNM person after disclosure Positive and supportive responses after disclosure Positive responses: from those who were young, progressive and had minority identities Rejection from personal contacts Assumptions of promiscuity
Structural barriers typically prevent disclosure	Fostering children IVF treatment Social welfare implications Maternity leave entitlements Impact on professional reputation Impact on employment Official documents are not adequate Erasure of partners
Unless specifically trained, healthcare providers are uninformed about CNM	Inadequately trained, lack of understanding Judgmental responding Ethics and integrity questioned CNM people seen as mentally unstable Hostility from healthcare providers Issues and assault blamed on CNM Silence and discomfort after disclosure Risk-based assumptions Dissuasion from STI testing Inadequate rapport Expectation for pathologization Inaccessibility of CNM trained providers Some willingness to learn and understand

CNM = consensual non-monogamy

### Theme 1: Decisions Around How and When to Disclose are Complex

The decision to disclose a non-monogamous orientation or relationship was found to be an ongoing, complex process involving debating the potential costs versus benefits of disclosure. The participants collectively described an ongoing oscillation between motivations to conceal their non-monogamy and a desire to openly disclose. These motivations comprised a combination of external pressures and internal drivers as detailed below. All participants except two revealed that they consistently made choices about disclosure in a selective fashion, and that the decision was dependent on the context and person disclosed to. The concealment of non-monogamy was valued for factors such as privacy, risk mitigation, and conservation of mental and emotional energy. Over half of the participants described their fears of the potential consequences of disclosure and subsequently chose not to disclose in some contexts to mitigate these risks. Importantly, this seemed to be more common and pertinent for participants from multicultural or culturally diverse backgrounds. Predicted risks included factors such as verbal and physical harassment, the impacts on work environments or legal standing, loss of friendships, negative impacts on wider family relationships, or having their non-monogamous identities taken as evidence of a pathology. One female participant who was ambiamorous (i.e., comfortable in both monogamous and non-monogamous relationships) and in an open relationship described disclosure as a risk for sexual microaggressions:^2^

I think being non-monogamous opens your up to being seen as promiscuous, and absolutely people assume that you’re sexually available. So, I often go dancing with my nesting partner’s metamour, and sure, when we dance we’re pretty touchy, but with each other. Anyway, when we meet people out, if we tell them about our relationship situations they always assume that we’ll be up for a threesome with them, and then they get really gross and won’t leave us alone… like they start touching us and won’t take no for an answer. It’s awful, it’s ruins the night… like, we’re just here to dance and have a good time–get away from me… yeah, it’s just so much easier not to tell people.

One-third of participants with a range of gender and relationship identities revealed that mental energy expenditure was a major factor in their decisions not to disclose. Some also described the deliberate consideration of predicting whether the benefits to disclosing would out-weigh the costs of the mental energy expenditure. A female who identified as a relationship anarchist discussed the potential mental energy costs of disclosure:

I think sometimes before having an interaction with someone, you do a quick calculation in your head of how much emotional labor or explanation you might need to provide the other person about being in an ethical non-monogamous relationship. If there isn’t much to be gained, especially for a short interaction then sometimes it’s just not worth the effort and it’s easier to be seen as either single or monogamous.

Disclosure was not seen as a one-off event but was rather viewed as a process that required energy and the possibility of ongoing, lengthy conversations to educate the people disclosed to about non-monogamy. Further, participants anticipated that disclosure could place stress on relationships and might consequently require further energy to rebuild and maintain their personal connections.

Conversely, despite the perceived negatives of disclosure, two-thirds of the sample described a strong desire to be open with their non-monogamy identities and relationship styles. Disclosure was valued for several factors, such as a sense of personal integrity and genuineness. A male participant who identified as a solo-polyamorist described the desire for the normalization of their relationship identity:

Honestly, for me it became a thing about just owning it, um, when I sort of realized that in a sense, that my truth was that I am who I am. Then it became a thing of just going, okay, well that means it should be no different to disclosing my age.

A further driver for disclosure included allowing partners to be introduced into wider social circles, thereby becoming more integrated into the participants’ lives. Accordingly, one female who identified as polyfidelitous described the importance of disclosure to ensure her partners could be introduced into her social circles:

As it became clear that we had partners that we wanted to be with for a long time, I wanted them to feel how I would feel if I was going into a monogamous relationship, like being able to meet parents at a normal time and go to parties and be invited to all of the things that you would do in a family. I didn't want these partners to feel like they were a secret or that there was anything to be ashamed of. So, um, when it became clear that these were real relationships, we came out to our families and that was hard, but yes, very open. So, we tell anyone and everyone it's on Facebook, we share photos, all of that kind of thing.

A further motivation for disclosure that emerged was a sense of responsibility to educate the wider public about non-monogamy, enhance representation, and ideally ease disclosure for those less able to safely disclose. One agender participant, who was committed to multiple intimate relationships, described their sense of responsibility to disclose non-monogamy:

I’m not in a high-risk category for discrimination… and I’ve also been more and more out about other aspects of my personal life in terms of like my gender identity at work and stuff and my disability and stuff like that… and having people respond really positively to me… has made me want to be more open about my relationships, and it’s partly a responsibility. Like a responsibility towards representation.

Finally, several participants felt motivated to disclose in order for personal contacts to understand the consensual and ethical nature of their various intimate relationships and to prevent these contacts from assuming infidelity. An agender participant in multiple committed intimate relationships explained their experience of this reason for openness of non-monogamy:

One of the reasons I did get more increasingly out at work is that I had the sense of, I didn’t want people to think I was cheating on any of my partners. So, if they had only met one of my partners and happened to see me out socially with another partner, what they’re...like, unfortunately more likely assume that I’m cheating than assume that I have like multiple relationships that everyone knows about.

### Theme 2: Responses to Disclosure are Typically Negative

Participants collectively described mixed responses to their disclosures of non-monogamy. A common theme that emerged was for personal and professional contacts to respond to disclosure with displays of discomfort. This was sometimes demonstrated when those who were disclosed to did not to respond at all, which was generally interpreted as stemming from discomfort and possibly from fear of saying the wrong thing. A female who identified as polyamorous and a relationship anarchist explained her experience after disclosing to her sister:

I noticed with my sister, she’s someone normally I can talk to about these kinds of things. And she was really weird about it. Like just didn't want to talk about it. I'd message her or something and she just wouldn't respond. So, it was almost like she just pretended it didn’t exist, which for me was really hurtful.

Experiences of rejection from family, friends, and employment settings as a result of stigma driven by relationship styles were very common. Indeed, every participant described either actual or anticipated rejection as a result of disclosing being consensually non-monogamous. One gender non-binary participant who identified as ethically non-monogamous described their experience of rejection from their family:

I told my parents, and they just, like, Mum just laughed and has never talked about either of my partners by name ever since, so they just don’t exist…Um, and my family kinda have started to boot me out a little bit, because of it. They’re also completely homophobic and racist so, ah, I’m the black sheep of that, yeah…I, I’m officially dating no one and my partners just don’t exist, which is really shit for them… Like, I wouldn’t be able to bring a partner to Christmas.

Other participants described how disclosures of non-monogamy were met with negativity that then often descended to negative comments about other aspects of their lives. A transfeminine polyamorous participant depicted how their first disclosure unfolded:

I told my family at our Chinese New Year celebrations that I had two partners, and, well, I guess I should have known better... My mom started ranting about how Western culture had ‘ruined me’ and that now ‘I had no values’. She said my gay friends were a bad influence and that if I’d been friends with the Chinese kids this wouldn’t have happened. I explained that my gay friends weren’t gay - that they were trans, and that this is different, but she screamed at me about how this is why I wasn’t doing well at uni and bunch of other stuff and then stormed off. My siblings were trying to comfort me but we could still hear my mum and her friends in the next room saying all sorts of horrible things about Aussies and how they don’t respect relationships – and … well more blame things about my gender journey [sic]. I wish I’d had the guts to tell them that the person who helped me understand on this open relationship stuff was Chinese too!

Other people being disclosed to demonstrated a lack of knowledge regarding how non-monogamous relationships work, for example, assuming that other partners would be unaware of the existence of each other. Other people being disclosed to conveyed their beliefs that non-monogamy was something that would be difficult to manage, or something that was unpleasant or even immoral. One male who identified as polyamorous described his experiences of friends responding to his disclosure with varied reactions including curiosity, distancing themselves from the idea of non-monogamous, and negative reactions about the morality of non-monogamy:

I mean then I guess you get a few friends that are kind of like quite intrigued by it and want to know what’s going on and you know. Um, there seems to be a general like common thread that a lot of people will be first of all, say, “Oh, I can never do that.” Um, or people sort of almost, you know, there’s a bit of resentful sort of thing. Maybe that sort of almost like this thing of, “Well, it’s a bit selfish” or “You’re having your cake and eating it” or something like that (sic).

Several participants revealed that they had been rejected by family and friends after disclosing their non-monogamous relationship styles, resulting in the loss of relationships. One female who identified as polyamorous described her friendship breakdown after disclosure:

I lost one very close friend over the disclosure. Um, I told her about the nature of my relationship with the married couple and she just couldn’t cope… there were worries about my wellbeing and how I was going to cope in the relationship. And then she was worried about their marriage and the impacts of our relationship on their marriage. And then they also had children. So then she was worried about the impact of our relationship on the children.

It is worth highlighting that negative experiences also contributed to some participants not wanting to disclose unless necessary. One female who identified as ethically non-monogamous described not wanting to “come out” as a result of second-hand experiences of stigmatization toward her non-monogamous friends:

I have friends who have been outed or who have come out and lost jobs or family or been harassed by people. I know people who aren’t invited to Christmas anymore… I don’t want that. I’ll keep my secret to myself, thanks.

Another polyamorous female participant described that in her Islamic cultural groups it would not have been safe for her to be openly non-monogamous:

I have to be very selective about who I tell–I have to keep it to people who I know won’t blab about it to my family. There’s so much gossip in our community–they thrive on it… so I really have to be very choosey [sic] about who gets to know. If I told the wrong person and it got, it would be a source of shame for my family, a real problem – they wouldn’t get it at all… I don’t think they’d disown me, but they really would see it that they have to “fix” the problem or “fix” me to get me “normal again” whatever that means. Ironically, I have a great uncle back home who has multiple wives… but I guess they would see it as different when it’s marriage and arranged, and it sure is different for men… eurch.^3^

Some participants conversely experienced neutral or even positive, supportive responses to disclosure of non-monogamy. Just less than half of the sample described at least one positive response to disclosure. About one-third of those (20% of the total sample) reported common positive experiences when disclosing. These participants described a similar characteristic of consciously surrounding themselves with people who were open-minded to different sets of life experiences and relationship situations, which they felt accounted for overall positive experiences. However, the majority of participants did not report being surrounded by non-monogamy friendly communities and were pleasantly surprised when met with supportive reactions. One female who identified as ethically non-monogamous described her positive experiences of disclosing to friends and family:

I told my family at Christmas just gone. Um, and they were really supportive…Yeah, they’ve been overwhelmingly positive. I told, there’s a bunch of friends who I haven’t seen in a while… and I told them everything that was happening. And, um, and they were just insanely curious. They were just, they’re all, you know, long-term married. So, you know, I was describing my life to them and I was like, you know, I think one of them said “You’re like a rockstar” (laughs).

Positive responses to non-monogamy disclosure were found to be more likely when the people being disclosed to were younger, more progressive, had more prior exposure to non-monogamy, or when the people disclosed to had other minority identities themselves (such as gender or sexual minority identities or disabilities). A gender-fluid participant identifying as a relationship anarchist described the ease of disclosing within their nerd and queer communities due to non-monogamy representation and awareness within these groups:

Yeah, telling friends about it at the start was pretty easy, um because of the community we were in, most of them had at least heard about it before and were seeing like other people with those patterns.

### Theme 3: Structural Barriers Typically Prevent Disclosure

Social and structural barriers were a common theme described by participants, involving societal obstacles which systemically excluded non-monogamous people. Every participant spoke about this theme with some form of encountered societal barriers. Participants described the many small barriers that were met on a daily basis. A polysexual non-monogamous man described:

Almost every day there’s some sort of reminder that my relationships aren’t normal and don’t’ fit the mould. Every time I fill in a form, it happens–it never allows me to describe my relationship properly. Same when you have to declare a next-of-kin, or want to submit an applications… there’s only ever room to describe one partner. And then, well it forces me to pick one, to prioritise one partner… we’ve all been in our relationship for nearly a decade, and it feels really bad to have to do that. We’ve talked about it, and they say that they understand, and that it’s just a form or whatever, but it doesn’t feel right. In these ways, I can’t show that I’m non-monogamous, even when I’m trying to!

In many instances, barriers and anticipated barriers prevented disclosure. For example, about a third of participants of varied genders spoke about the added complications and impetus to conceal their non-monogamous relationships when considering fostering children or seeking IVF treatments. One female who identified as monogamish explained her fear that she would be excluded from fostering if her relationship identity was known:

I don’t plan on coming out as ethically non-monogamous. Um…I’m planning on fostering, and because of the stories I’ve heard about people who have multiple partners being not allowed to foster, I don’t plan on coming out in the foster care system.

Participants also spoke about the financial implications for being in non-monogamous relationships, such as repercussions with welfare payments and maternity leave entitlements. One female participant who identified as ethically non-monogamous illustrated this with her explanation that she felt non-monogamy was excluded from the current societal model of leave and welfare entitlements:

It's really difficult cause our entire society is built on the idea of monogamy. Um, so, you know, um, things like social welfare and, um, you know, um, maternity leave and stuff like that, you know, it's all based on this monogamous, “man woman living together with kids” kind of idea.

One male who identified as a relationship anarchist explained his fear of being financially penalized for disclosing their non-monogamy to their local welfare office. He described the assumptions within social welfare that having romantic relationships meant a change to an individual’s level of social support, but explained that non-monogamous relationships did not always follow this structure of resource sharing between partners:

According to Centrelink (local welfare office), I always, or I pretty much always will be single or divorced because my version of partner is not the heteronormative one of sharing a house and, and sharing bills and things like that… They don't have a space on their forms and… I don't think they have space in the regulations for multiple ethical connections at the same time without it being impacted, financially impacted.

Finally, participants shared their fears of losing their jobs, having disclosure impact on their professional reputations, or opening themselves up to stigmatizing reactions. One female who identified as polyamorous and a relationship anarchist explained her fear for her professional reputation and employment were her non-monogamy to be known:

I wouldn't want to risk losing my job or anything like that. Especially with the working with children's check… And although I haven't experienced it first hand, it's just a fear that’s there, if that makes sense.

Another female participant who identified as ethically non-monogamous explained her fear for her professional reputation and employment were her non-monogamy disclosed:

I'm also interested in politics, but I have made the decision that I would never pursue that because of non-monogamy…I wouldn't hide it. I think it would be too difficult because of the attitudes that Australia has towards female politicians full stop. But also, to this idea of what is appropriate sexual behavior and, you know, it's, uh, it's… Australia is just not ready I think. And I wouldn't want to be exposed to all of the, all of that bullshit, and slander and stuff. It's just not worth it. But it's sad because I'm sure that plenty of other people in [the] non-monogamous community would feel that way as well. Which means that we're not going to be represented.

### Theme 4: Unless Specifically Trained, Healthcare Providers Are Typically Uninformed About Non-monogamy

Responses to disclosure of non-monogamy from healthcare providers were discussed often in the data. Almost every participant had a story, although they varied in nature. Some participants described that healthcare providers responded to disclosure in an inadequate, judgmental, or even stigmatizing manner, while others responded in a manner deemed as adequate or even supportive. However, the most common theme that emerged was for healthcare providers to respond to disclosure by lapsing into an uncomfortable silence or ignoring the disclosure altogether, with over half of the sample reporting multiple experiences where practitioners responded to disclosure of non-monogamous relationship identities in a manner perceived as visible discomfort. One gender-fluid participant who identified as a relationship anarchist described their general experience of silence from doctors in response to disclosure of non-monogamy, which was perceived as indifference or discomfort:

I have had professionals at other clinics, where they’ll ask those questions and I’ll answer them, and they just have nothing to say afterwards, and like continue to move on, and like it’s clear that they don’t want to say the wrong thing, but they don’t have a response or know how to…um, which isn’t awful but it’s not comfortable.

Several participants spoke of healthcare professionals who responded to their disclosure of non-monogamy with lecturing and risk-based assumptions, leaving the participants feeling misunderstood, judged, and demeaned. Over half of the sample described experiences of stigma from healthcare practitioners on at least one, if not several occasions. A gender non-binary person identifying as ethically non-monogamous described their experience of feeling judged by a general practitioner, when the doctor found out about their consensually non-monogamous identity and immediately asked questions about STI transmission, despite sex not being relevant to their reason for presenting:

So, with the GP, I… so I think I was, something really, really basic, um… and like I probably needed a script for something… and I, I just let it slip… I just said yeah “One of my partners is picking me up” or something… And that was like, “Oh so… what did you say?” and I’m like “Yeah sorry, one of my partners, I have two partners”, and um, and then all of a sudden they started talking to me about STI’s, and they were asking me questions around “Have you ever had Chlamydia?”, like that kind of thing. And um, it was really insulting. I was like, yep, it just demeaned me as a person. It like…the assumption to them was that I would have an STI because I have two partners.

In fact, more than half of the participants discussed either directly experiencing stigmatization from healthcare professionals including nurses, GPs, and mental health practitioners, or having a partner or non-monogamous close friend who were stigmatized as a result of their identities. One male who identified as solo-polyamorous described their experience of judgment and stigmatization from prior therapists:

Um, the, I had a situation with two previous therapists… Um, one, she tries to be neutral about it, but you could see, um, that she was biased against ethical non-monogamy, um, and the other one was outright hostile against it… She turned around and went “Ach! You polyamorous people are all the same, all you wanna do is just fuck, fuck, fuck.” And I went “Oh! I got it. Bye bye”.

There was a common thread of practitioners’ lack of awareness and education regarding this population which presented difficulties in providing adequate health care. This included issues such as health practitioners talking people out of STI testing, with over one quarter of the participants describing difficulties in acquiring judgment-free STI testing or testing with the frequency that they desired. Despite perceived negative experiences, 27 out of 32 participants spoke about understanding the importance of openness with healthcare professionals in order to ensure adequate sexual health care and also to build understanding and rapport, especially with mental health practitioners. A female identifying as ethically non-monogamous explained her general experiences with healthcare professionals who she felt were not adequately addressing her sexual healthcare needs:

I generally get the response that I don't need to have HIV testing because I'm a woman. And I find that really a little bit negligent… they’re just ignoring the fact that I could be involved in group sex with strangers. And that I date guys that are bisexual as well. I've never had a GP offer me a throat swab.

Similarly, another female participant who identified as ethically non-monogamous described how she was talked out of regular STI testing by a GP:

I have been questioned once by one GP, who was not my usual GP. And he was a little bit, you know, “Why are you getting this done so often? Aren't you using protection?”…Yeah, the implication was that, um, if I was using protection and if I was, you know, if I had a good safer sex strategy, then…um, then I shouldn't have to keep getting these STI tests.

In several cases, participants anticipated that health practitioners would pathologize their non-monogamy and assume causation of non-monogamy to unrelated health issues due to the experiences of their close personal contacts. One example of this was given by an agender participant who was in multiple committed intimate relationships:

I’ve had so many friends and partners and people who have just had really shit experiences in counselling where they’ve just had everything blamed on the fact that they’re non-monogamous. Their mental health, their poor relationship with their narcissist of a mother, their lack of self-esteem… everything!

A gender non-binary participant identifying as ethically non-monogamous described their difficult experience, where they felt victim blamed by a therapist, who assumed their non-monogamous identity was the causal factor in their sexual assault:

And I had a counsellor who I was talking to about how I was raped a couple of times, um, and it came out that I was non-monogamous and so she was like, and, so it was my ex-husband that raped me, and… she was like “Ohh so was your husband not happy that you had another partner?” And I was like, “So are you now blaming me, for...are you saying that because I was non-monogamous that that’s why I was raped?” and she just kind of sat there in silence and I was like “You need to choose your words very carefully right now”. And then I became the counsellor and I came, ah, had to educate her on how it wasn’t non-monogamy that caused rape, it was a like unstable abused husband as a child that caused rape.

Just under half of the sample reported experiencing a positive response to disclosure of non-monogamy from at least one healthcare professional, with several of these participants stating that they specifically chose healthcare professionals who had been recommended to them or were believed to be friendly or affirming of non-monogamy. Healthcare providers in specific workplaces or ones with specialized training who advertised their awareness and acceptance of non-monogamous communities were found to be very helpful and well educated regarding the healthcare needs of minority populations. However, these services were not highly accessible due to factors such as location or long waiting lists. This experience was described by a gender non-binary person identifying as ethically non-monogamous:

Like I’ve got, I’ve got a friend who is a non-monogamous LGBTQIA kink positive counsellor, and she has been booked out with a six to eight month waiting list for a year and a half. Because no one has anyone to go to, and it’s really fucked.

Other participants described how LGBTQ-specific service providers could be assumed to be synonymous with being non-monogamy friendly. A polyamorous man described:

I’m not gay, but I will go to a gay service again in a heartbeat. I went to a doctor for gay people with one of my girlfriends, about sexual health and we needed some kind of counselling about our relationships. It took a minute to get over myself and open up… but once I did, it was the best! They treated us like our relationships were normal… even better, like better than normal! Like, there were no questions about it, and they… well, they just got it!

Two participants reported some positive experiences with health professionals who were supportive and non-judgmental. These practitioners were reported to have taken the time to educate themselves and understand how to enhance their supportiveness of non-monogamous people, including the related healthcare implications. One of these participants was a female identifying as polyamorous. She described her positive experience with a health practitioner who treated her non-judgmentally despite not having previous experience with non-monogamous patients:

I never felt from my allied health professional that he wasn’t all over what I was talking with him about… like he hasn't had clients coming in in those similar kinds of relationships… But, it was also great that he was very accepting and very nonjudgmental. Um, yeah, it was brilliant.

Similarly, a male who described himself as hierarchically ethically non-monogamous described a similar experience with his GP who took it upon herself to educate herself about non-monogamy:

The GP that we see… we lucked out. She’s very understanding of it, she, I don’t think that she understood it in the sense that she had other people in the same situation, but she could… yeah, she asked for book recommendations as well, so I recommended one or two. Um, yeah so, the GP took it very, very well and she tried to learn more about it, and because she’s also working on the mental health side of things, she was kind of staying on top of it in terms of how it affected us in terms of psychological help.

Overall, almost half of the total sample stated that they specifically sought out healthcare professionals who had prior non-monogamy awareness in order to avoid negative encounters during healthcare visits or to avoid having to spend their session time educating practitioners. The same proportion of participants discussed their strong desires for an increase in non-monogamy education among healthcare professionals.

## Discussion

This study explored the experiences and consequences of disclosure for non-monogamous people. The results suggested that while non-monogamous people desire openness, they often conceal their relationship identities for a range of factors. These include issues such as concern for reputational risk and employment insecurity within workplace environments, the effort and energy required in explaining non-monogamy to others due to a general lack of awareness and representation of non-monogamy, fear of stigma, and fear of repercussions such as reductions in welfare support or being denied rights to foster children. Participants further described the persistent discomfort or lack of understanding they encountered from personal contacts and healthcare professionals alike after disclosing their non-monogamous identities or relationships. Finally, repercussions of disclosure were discussed, such as the pathologization of relationship identities within healthcare settings, assumed and experienced stigmatization and rejection by personal contacts. The impacts of stigma and structural barriers to disclosure appeared to be consistent across the sample, regardless of relationship identity or other demographic characteristics. The experience of inadequate education regarding non-monogamy among healthcare professionals was widespread throughout the data and clearly indicated a need for increased representation, awareness, and education of non-monogamous relationship identities among practitioners. Future research is required to examine the mental and physical health implications of inadequate education across healthcare settings for non-monogamous populations.

It is important to note that misunderstanding, negative reactions, and prejudiced attitudes and behaviors were prevalent in the disclosure narratives provided by the participants. This aligns with existing evidence which suggests that non-monogamous individuals often feel stigmatized and experience discrimination on the basis of their identity or relationship configurations (Burleigh et al., 2017; Thompson et al., 2020; Vil et al., 2022), and certainly our participants described the effects of mononormativity in their experiences of disclosure. Participants discussed many ways that this antipathy manifested in response to disclosures, such as explicit experiences of abuse and maltreatment (physical and psychological) but also in the form of anticipated discrimination which resulted in considerable emotional labor. Of note, there was a paradox in the narratives of our participants, in which they described being both overly sexualized by medical professionals and also policed around their sexual health (e.g., denial of access to STI testing). While speculative, it is worth considering the possibility that this contradiction could be a form of punishing non-mononormative relationships.

While not the focus of this study, intersectionality (an overlapping repression of power due to multiple marginalized identities; Crenshaw, 2017) clearly emerged as a strong pattern across the data and is an important area of study for future research. All participants in this study identified with a sexuality other than heterosexual, and more than a quarter of the sample identified outside of the male/female gender binary. Consequently, each participant was likely to have had past experiences of disclosing a minority identity, which likely colored their expectations and perceptions when considering disclosure of non-monogamy. This was particularly the case as most participants described their non-monogamous identities as evolving fairly recently (generally within the past 2 to 10 years), mostly after sexual and gender identities were established.

In addition, there were a range of other cultural and religious identities present in the sample and each of these additional intersecting identities complicated the disclosure process. In particular, the participants from the minoritized cultural and religious backgrounds (which in Australia is religions other than Christianity, and ethnicities other than White) reported more frequent and more intense negative experiences with non-monogamy disclosure. This aligns well with other research that discusses “coming out” as being a privilege that not all individuals are afforded. For a range of reasons, individuals in specific cultures or with specific religious backgrounds might not wish to come out about their non-monogamy, and “inviting in” might be a more appropriate manner for disclosures about non-monogamous identities or relationships (for a discussion on “inviting in,” see Hammoud-Beckett, 2007, 2022).

Of the 32 participants, only eight identified with a religion and another nine identified as being spiritual—the remaining participants stated they were not religious or did not have a religion, or they identified as atheist. This may result from a selection bias, or it may indicate an underlying lack of compatibility between religion and non-monogamy. It is a distinct possibility that disclosure for non-monogamous religious people could present unique challenges, such as fear of rejection from their communities, and this subject could benefit from further examination (for work on religious non-compatible identities, see Anderson et al., 2023).

### Implications and Limitations

There are a range of implications from the findings of this study. Foremost, it is clear that there is a need for increased knowledge around non-monogamy (for the general public, but also for service providers). Prejudice and stigma experienced by non-monogamous individuals is likely to be damaging to their relationships and their well-being and will discourage disclosures around non-monogamous identities and relationship structures. This has a range of implications for poorer well-being based on living life inauthentically, discouraging open communication, and straining familial relations, and there are additional negative impacts for any partners who are impacted by non-disclosure (see Balzarini & Muise, 2020). In addition to the social implications, there are a range of implications for service providers. For instance, specialist training in non-monogamy-related issues should be included for healthcare workers in order to best serve the needs of this population. A concurrent or alternative solution could include a centralized search function to easily identify healthcare practitioners with non-monogamy awareness training for use by the non-monogamous population and by referring practitioners. This study highlighted a need for stronger inclusion and awareness of non-monogamous people within social systems, such as healthcare settings, social welfare, and administrative forms in general. Furthermore, there is an urgent need to consider how non-monogamy is considered in sexual health, ranging right from sex education through to applications such as STI screening. Finally, research regarding the mental health impacts of ongoing concealment of non-monogamy would be beneficial for the future of research in this field.

Several limitations impacted on the generalizability of the study results. The sample was drawn mostly from non-monogamous people who identified as polyamorous or relationship anarchists and did not include other identities, whose disclosure experiences may have differed significantly from this sample. Put simply, the non-monogamy being discussed by the participants was more about relationships and dating than sexual non-monogamy (although of course there is overlap). Despite efforts to recruit diversely, we acknowledge that we did not have a range of representation across many relevant dimensions, including socio-economic status, sexuality, political orientation, or ability status. Future samples should aim to contain a representative range of demographic characteristics, including a broader range of non-monogamous relationship identities. We also acknowledge that the Australian context in which this study was conducted might have impacted the findings of the study—there is some evidence to suggest that Australia is relatively open to non-conventional relationship structures (at least compared to some other parts of the world; Hosking, 2014), and our participants were mostly based on very metropolitan areas, which are typically more progressive than other areas. Our sample was all relatively ‘out’ as non-monogamous (with some exceptions) and had self-selected to participate (e.g., through advertisement on non-monogamy social media groups), and so their experiences of disclosure are not likely to be reflective of all experiences of disclosure.

As previously stated, the current sample also had a number of intersections which were likely to impact on their experiences of coming out. Furthermore, the interviews were all conducted as the COVID-19 pandemic was spreading throughout the globe, and may have impacted on participants’ levels of stress or mental space to be able to consider this topic in adequate depth. There may also have been practical implications due to participants’ reduced interactions with social contacts and colleagues, rendering contemporaneous experiences of disclosure less likely. Further to this, participants’ relationships might have been placed under increased pressure due to the restrictions which barred them from seeing more than one intimate partner. This may have resulted in additional stress due to forced prioritization of partners and perhaps a resultant change in their relationship conceptualizations.

### Conclusion

This study sought to examine the experiences and consequences of disclosure for non-monogamous people and found that people with a non-monogamous identity experience typically negative responses to disclosure. Personal contacts of non-monogamous people commonly did not know how to respond to disclosure and sometimes responded with extensive questioning, rejection, and stigmatization of these relationship identities. Consensually non-monogamous people consequently often chose to disclose selectively or to keep their relationship identities concealed. Healthcare providers were frequently found to be lacking in the experience and cultural awareness to work effectively with this community. Finally, consensually non-monogamous people were found to be marginalized by their overarching lack of representation and inclusion within the core structures of society.

## Supplementary Information

Below is the link to the electronic supplementary material.

Supplementary file1 (DOCX 12 kb)

## Data Availability

Data can be requested by contacting the corresponding author (joel.anderson@latrobe.edu.au).

## Footnotes

1 Please note that, in Australia, male and female refer to the participants’ gender identities rather than sex markers (ACON, 2023).

2 A nesting partner refers to a partner that someone lives with. A metamour refers to the partner of one's partner (where there is no direct romantic or sexual relationship).

3 This participant used double quotation air gestures when saying the word “fix.”

## References

[CR1] ACON. (2023). *ACON recommended community indicators for research*. https://www.acon.org.au/wp-content/uploads/2016/03/ACON-Recommended-Community-Indicators-for-Research.jpg.

[CR2] Anderson, J. R., Hinton, J. D. X., Bondarchuk-McLaughlin, A. Rosa, S., & Tan, K. J. (2025). Countering the monogamy-superiority myth: A systematic review and meta-analysis of the differences in relationship satisfaction and sexual satisfaction as a function of relationship orientation. *Journal of Sex Research*. 10.1080/00224499.2025.2462988 40126203 10.1080/00224499.2025.2462988

[CR3] Anderson, J. R., Kiernan, E., & Koc, Y. (2023). The protective role of identity integration against internalized sexual prejudice for religious gay men. *Psychology of Religion and Spirituality,**15*(3), 379–388. 10.1037/rel0000452

[CR4] Balzarini, R. N., & Muise, A. (2020). Beyond the dyad: A review of the novel insights gained from studying consensual non-monogamy. *Current Sexual Health Reports,**12*, 398–404. 10.1007/s11930-020-00297-x

[CR5] Barker, M., & Langdridge, D. (2010). *Understanding non-monogamies*. Routledge.

[CR7] Brewster, M. E., Soderstrom, B., Esposito, J., Breslow, A., Sawyer, J., Geiger, E., Morshedian, N., Arango, S., Caso, T., Foster, A., Sandil, R. K., & Cheng, J. (2017). A content analysis of scholarship on consensual nonmonogamies: Methodological roadmaps, current themes, and directions for future research. *Couple and Family Psychology: Research and Practice,**6*, 32–47. 10.1037/cfp0000074

[CR8] Burleigh, T. J., Rubel, A. N., & Meegan, D. V. (2017). Wanting ‘The Whole Loaf’: Zero-sum thinking about love is associated with prejudice against consensual non-monogamists. *Psychology & Sexuality,**8*, 24–40. 10.1080/19419899.2016.1269020

[CR9] Campbell, J. L., Quincy, C., Osserman, J., & Pedersen, O. K. (2013). Coding in-depth semistructured interviews: Problems of unitization and intercoder reliability and agreement. *Sociological Methods & Research,**42*, 294–320. 10.1177/0049124113500475

[CR10] Cardoso, D., Pascoal, P. M., & Maiochi, F. H. (2021). Defining polyamory: A thematic analysis of lay people’s definitions. *Archives of Sexual Behavior,**50*(4), 1239–1252. 10.1007/s10508-021-02002-y 34046765 10.1007/s10508-021-02002-y PMC8321986

[CR11] Conley, T. D., Moors, A. C., Matsick, J. L., & Ziegler, A. (2013a). The fewer the merrier?: Assessing stigma surrounding consensually non-monogamous romantic relationships. *Analyses of Social Issues and Public Policy,**13*(1), 1–30. 10.1111/j.1530-2415.2012.01286.x

[CR12] Conley, T. D., & Piemonte, J. L. (2021). Are there “better” and “worse” ways to be consensually non-monogamous (CNM)?: CNM types and CNM-specific predictors of dyadic adjustment. *Archives of Sexual Behavior,**50*(4), 1273–1286. 10.1007/s10508-021-02027-3 34100142 10.1007/s10508-021-02027-3

[CR13] Conley, T. D., Ziegler, A., Moors, A. C., Matsick, J. L., & Valentine, B. A. (2013b). A critical examination of popular assumptions about the benefits and outcomes of monogamous relationships. *Personality and Social Psychology Review,**17*(2), 124–141. 10.1177/1088868312467087 23175520 10.1177/1088868312467087

[CR14] Crenshaw, K. W. (2017). *On intersectionality: Essential writings*. The New Press.

[CR15] Flicker, S. M., Sancier-Barbosa, F., Moors, A. C., & Browne, L. (2021). A closer look at relationship structures: Relationship satisfaction and attachment among people who practice hierarchical and non-hierarchical polyamory. *Archives of Sexual Behavior*, *50*, 1401–1417. 10.1007/s10508-020-01875-9 33956295 10.1007/s10508-020-01875-9

[CR16] Forrester, M. A., & Sullivan, C. (2018). *Doing qualitative research in psychology: A practical guide*. SAGE Publications Limited.

[CR17] Gupta, S., Tarantino, M., & Sanner, C. (2024). A scoping review of research on polyamory and consensual non-monogamy: Implications for a more inclusive family science. *Journal of Family Theory & Review*, *16*, 151–190. 10.1111/jftr.12546

[CR18] Hamilton, L. D., De Santis, C., & Thompson, A. E. (2021). Introduction to the special section on consensual non-monogamy. *Archives of Sexual Behavior,**50*(4), 1217–1223. 10.1007/s10508-021-02055-z 34089124 10.1007/s10508-021-02055-z

[CR100] Hammoud-Beckett, S. (2007). Azima ila Hayati-An invitation in to my life: Narrative conversations about sexual identity. *International Journal of Narrative Therapy & Community Work*, *1*, 29–39.

[CR19] Hammoud-Beckett, S. (2022). Intersectional narrative practice with queer muslim clients. *Journal of Intercultural Studies,**43*(1), 120–147. 10.1080/07256868.2022.2016664

[CR130] Hosking, W. (2013). Satisfaction with open sexual agreements in Australian gay men’s relationships: The role of perceived discrepancies in benefit. *Archives of Sexual Behavior*, *42*, 1309–1317. 10.1007/s10508-012-0005-9 23001498 10.1007/s10508-012-0005-9

[CR20] Hosking, W. (2014). Australian gay men’s satisfaction with sexual agreements: The roles of relationship quality, jealousy, and monogamy attitudes. *Archives of Sexual Behavior,**43*, 823–832. 10.1007/s10508-013-0197-7 24287963 10.1007/s10508-013-0197-7

[CR21] Hutzler, K. T., Giuliano, T. A., Herselman, J. R., & Johnson, S. M. (2016). Three’s a crowd: Public awareness and (mis) perceptions of polyamory. *Psychology & Sexuality,**7*(2), 69–87. 10.1080/19419899.2015.1004102

[CR22] Klesse, C. (2006). Polyamory and its ‘others’: Contesting the terms of non-monogamy. *Sexualities,**9*(5), 565–583. 10.1177/1363460706069986

[CR24] Levine, E. C., Herbenick, D., Martinez, O., Fu, T.-C., & Dodge, B. (2018). Open relationships, nonconsensual nonmonogamy, and monogamy among U.S. adults: Findings from the 2012 National Survey of Sexual Health and Behavior. *Archives of Sexual Behavior,**47*(5), 1439–1450. 10.1007/s10508-018-1178-7 29696552 10.1007/s10508-018-1178-7 PMC5958351

[CR25] Manley, M. H., Legge, M. M., Flanders, C. E., Goldberg, A. E., & Ross, L. E. (2018). Consensual nonmonogamy in pregnancy and parenthood: Experiences of bisexual and plurisexual women with different-gender partners. *Journal of Sex & Marital Therapy*. 10.1080/0092623X.2018.1462277 10.1080/0092623X.2018.1462277 PMC6185819 29648961

[CR27] McCrosky, R. (2015). *Experiences of stigma during sexual healthcare visits: A qualitative study of non-monogamous women.* Master's thesis. Retrieved from STARS Electronic Theses and Dissertations database. (Identifier No. CFE0005662).

[CR28] Meyer, I. H. (2003). Prejudice, social stress, and mental health in lesbian, gay, and bisexual populations: Conceptual issues and research evidence. *Psychological Bulletin,**129*(5), 674–697. 10.1037/0033-2909.129.5.674 12956539 10.1037/0033-2909.129.5.674 PMC2072932

[CR29] Meyer, I. H., & Frost, D. M. (2013). Minority stress and the health of sexual minorities. In C. J. Patterson & A. R. D’Augelli (Eds.), *Handbook of psychology and sexual orientation* (pp. 252–266). Oxford University Press.

[CR30] Moors, A. C., Gesselman, A. N., & Garcia, J. R. (2021a). Desire, familiarity, and engagement in polyamory: Results from a national sample of single adults in the United States. *Frontiers in Psychology,**12*, 619640. 10.3389/fpsyg.2021.619640 33833712 10.3389/fpsyg.2021.619640 PMC8023325

[CR31] Moors, A. C., Schechinger, H. A., Balzarini, R., & Flicker, S. (2021b). Internalized consensual non-monogamy negativity and relationship quality among people engaged in polyamory, swinging, and open relationships. *Archives of Sexual Behavior,**50*(4), 1389–1400. 10.1007/s10508-020-01885-7 34100145 10.1007/s10508-020-01885-7

[CR110] Moors, A. C., Selterman, D. F., & Conley, T. D. (2017). Personality correlates of desire to engage in consensual non-monogamy among lesbian, gay, and bisexual individuals. *Journal of Bisexuality*, *17*(4), 418–434. 10.1080/15299716.2017.1367982

[CR120] Nguyen, J., Anderson, J. R., & Pepping, C. A. (2023). A systematic review and research agenda of internalized sexual stigma in sexual minority individuals: Evidence from longitudinal and intervention studies. *Clinical Psychology Review*, *108*. 10.1016/j.cpr.2023.102376 10.1016/j.cpr.2023.102376 38218122

[CR32] O’Connor, C., & Joffe, H. (2020). Intercoder reliability in qualitative research: Debates and practical guidelines. *International Journal of Qualitative Methods,**19*, 1609406919899220.

[CR33] Rubel, A. N., & Burleigh, T. J. (2020). Counting polyamorists who count: Prevalence and definitions of an under-researched form of consensual nonmonogamy. *Sexualities,**23*(1–2), 3–27. 10.1177/1363460718779781

[CR34] Scoats, R., & Campbell, C. (2022). What do we know about consensual non-monogamy? *Current Opinion in Psychology,**48*, 101468. 10.1016/j.copsyc.2022.101468 36215906 10.1016/j.copsyc.2022.101468

[CR35] Sheff, E. A., (2019). Updated estimate of number of non-monogamous people in U.S.: Here are surprising results from recent research. *Psychology Today*. https://www.psychologytoday.com/au/blog/the-polyamorists-next-door/201905/updatedestimate-number-non-monogamous-people-in-us.

[CR36] Smith, L. J. (2017). *A qualitative study of the healthcare experiences of consensually nonmonogamous adults.* Unpublished Master’s dissertation, University of Washington. Retrieved from https://digital.lib.washington.edu/researchworks/bitstream/handle/1773/40595/Smith_washington_0250O_17803.pdf?sequence=1&isAllowed=y.

[CR37] Solutions, Q. (2001). *NVivo qualitative data analysis programme version 1.3 [Computer software]*. QSR International Pty Ltd Victoria.

[CR38] *The Adoption Act 1984* (Vic). http://classic.austlii.edu.au/au/legis/vic/consol_act/aa1984107/.

[CR40] Thompson, A. E., Moore, E. A., Haedtke, K., & Karst, A. T. (2020). Assessing implicit associations with consensual non-monogamy among U.S. early emerging adults: An application of the single-target Implicit Association Test. *Archives of Sexual Behavior,**49*, 2813–2828. 10.1007/s10508-020-01625-x 32297098 10.1007/s10508-020-01625-x

[CR41] Valadez, A. M., Rohde, J., Tessler, J., & Beals, K. (2020). Perceived stigmatization and disclosure among individuals in consensually nonmonogamous relationships. *Analyses of Social Issues and Public Policy*, *20*, 143–165. 10.1111/asap.12194

[CR42] Vaughan, M. D., Jones, P., Taylor, B. A., & Roush, J. (2019). Healthcare experiences and needs of consensually non-monogamous people: Results from a focus group study. *Journal of Sexual Medicine,**16*(1), 42–51. 10.1016/j.jsxm.2018.11.006 30621924 10.1016/j.jsxm.2018.11.006

[CR43] Vil, N. M. S., Bay-Cheng, L. Y., Ginn, H. G., & Chen, Z. (2022). Perceptions of monogamy, nonconsensual nonmonogamy and consensual nonmonogamy at the intersections of race and gender. *Culture, Health & Sexuality,**24*(1), 109–124. 10.1080/13691058.2020.1817561 10.1080/13691058.2020.1817561 32996411

[CR45] Witherspoon, R. G., & Theodore, P. S. (2021). Exploring minority stress and resilience in a polyamorous sample. *Archives of Sexual Behavior,**50*(4), 1367–1388. 10.1007/s10508-021-01995-w 34109526 10.1007/s10508-021-01995-w

