# Supplementary material for: A Qualitative Exploration of the Experiences of Disclosing Non-Monogamy
Source: Arch Sex Behav. 2025 Mar 24;54(4):1481–95. doi: 10.1007/s10508-025-03119-0 (PMC12011937; doi:10.1007/s10508-025-03119-0)
Supplement: Supplementary file 1 — Supplementary file1 (DOCX 12 kb) [file 10508_2025_3119_MOESM1_ESM.docx]

**Glossary**

**Primary partner or primary relationship:** Two people in a romantic relationship where their partnership is hierarchical, or esteemed above other intimate connections. Primary partners may choose to merge their lives and share resources via financial and residential arrangements.

**Hierarchical / hierarchically ethically non-monogamous:** A person who values or has a primary relationship that is esteemed above other intimate connections but may be open to engaging in romantic and or sexual connections with others. Romantic connections may be ranked as primary and possibly as secondary etcetera in this style of relating.

**Monogamish:** A person who is mostly monogamous or mostly desiring of monogamous relationships, but open to some negotiation around intimate relating outside of a primary relationship.

**Solo-polyamory:** A person who views themselves as their primary partner, or alternatively a person who relates intimately with others without a primary partnership or hierarchical ranking of partners.

**Relationship anarchy:** This form of relating holds a belief that all relationships (romantic or otherwise) should be consciously formed according to the boundaries and agreements of the people inside those relationships and not bound by social expectations.

**Polyfidelitous:** This is a style of romantic relating in which a core group of more than two consenting people form an intimate relationship that is closed to intimate connection with outside partners.
